# Abundant and Rare Microbial Biospheres Respond Differently to Environmental and Spatial Factors in Tibetan Hot Springs

**DOI:** 10.3389/fmicb.2018.02096

**Published:** 2018-09-19

**Authors:** Yanmin Zhang, Geng Wu, Hongchen Jiang, Jian Yang, Weiyu She, Inayat Khan, Wenjun Li

**Affiliations:** ^1^State Key Laboratory of Biogeology and Environmental Geology, China University of Geosciences, Wuhan, China; ^2^State Key Laboratory of Biocontrol and Guangdong Provincial Key Laboratory of Plant Resources, School of Life Sciences, Sun Yat-sen University, Guangzhou, China

**Keywords:** Tibetan hot springs, abundant biosphere, rare biosphere, environment factors, spatial factors

## Abstract

Little is known about the distribution and ecological functions of abundant, intermediate, and rare biospheres and their correlations with environmental factors in hot springs. Here, we explored the microbial community composition of total, abundant, intermediate, and rare biospheres in 66 Tibetan hot springs (pairwise geographic distance 0–610 km, temperature 32–86°C, pH 3.0–9.5, and salinity 0.13–1.32 g/L) with the use of Illumina MiSeq high-throughput sequencing. The results showed that the abundant sub-communities were mainly composed of *Chloroflexi, Proteobacteria, Deinococcus-Thermus, Aquificae, Bacteroidetes*, and *Firmicutes*. In contrast, the rare sub-communities mainly consisted of most newly proposed or candidate phyla of *Dictyoglomi, Hydrogenedentes, Atribacteria, Hadesarchaea, Aminicenantes, Microgenomates, Calescamantes, Omnitrophica, Altiarchaeales*, and *Chlamydiae*. However, the abundant and rare sub-communities shared some common phyla (e.g., *Crenarchaeota, Bathyarchaeota*, and *Chlorobi*), which were composed of different OTUs. The abundant, intermediate, and rare sub-communities were mainly influenced by different environmental variables, which could be ascribed to the fact that they may have different growth and activity and thus respond differently to these variables. Spatial factors showed more contribution to shaping of the intermediate and rare communities than to abundant sub-community, suggesting that the abundant taxa were more easily dispersed than their rare counterparts among hot springs. Microbial ecological function prediction revealed that the abundant and rare sub-communities responded differently to the measured environmental factors, suggesting they may occupy different ecological niches in hot springs. The rare sub-communities may play more important roles in organic matter degradation than their abundant counterparts in hot springs. Collectively, this study provides a better understanding on the microbial community structure and potential ecological functions of the abundant and rare biospheres in hot spring ecosystems. The identified rare taxa provide new opportunities of ecological, taxonomic and genomic discoveries in Tibetan hot springs.

## Introduction

Microbes are the dominant life form in hot springs, and their community compositions are commonly linked with ecological functions. Therefore investigation of microbial community composition and their metabolic functions is of great importance to understanding of biogeochemical cycling of carbon, nitrogen and sulfur elements in geothermal environments (Rothschild and Mancinelli, 2001; Fuhrman, 2009). So far a large number of microbial investigations have been performed in hot springs worldwide, such as Yellowstone National Park (Meyer-Dombard et al., 2005) and the Great Basin in the United States (Costa et al., 2009), Kamchatka in Russia (Kublanov et al., 2009), Malaysian (Chan et al., 2015), Yunnan (Hou et al., 2013), and Tibetan Plateau in China (Lau et al., 2009; Song et al., 2010, 2013; Huang et al., 2011; Wang et al., 2013). Among some of these previous studies, temperature was shown more important in shaping microbial community than other environmental parameters (e.g., pH and water chemistry) and spatial factors (biogeography) (Skirnisdottir et al., 2000; Pearson et al., 2008; Hou et al., 2013; Wang et al., 2013; Saxena et al., 2017). However, the microbial diversity did not show a monotonic relationship with temperature, suggesting other environmental variables and/or spatial factors may also jointly shape the microbial community (Yim et al., 2006; Purcell et al., 2007). For example, microbial communities were dominated by different phylogenetic groups in the low- and high-sulfide hot spring mats with same temperature (Skirnisdottir et al., 2000); and hyperthermophilic archaeal communities showed geographical distribution pattern in various hot springs (Whitaker et al., 2003). Thus it can be seen that environmental and spatial variables are two types of important factors shaping microbial community compositions in natural ecosystems (Nemergut et al., 2013). However, few researches have ever investigated the relative contribution of environmental and spatial factors to shaping of microbial communities in hot springs.

Considering the contribution of different microbial species to the diversity and biomass in ecosystems, microbial communities could be classified into abundant and rare (relative abundance >1 and <0.01%, respectively) taxa, with the former contributing major biomass but minor biodiversity and the latter contributing minor biomass but major biodiversity (Galand et al., 2009; Pedrós-Alió, 2012). Abundant taxa were the basis of most of our up-to-date knowledge on microbial diversity and response to environmental and spatial factors in hot springs, while rare taxa were usually treated as analytical annoyance (Jousset et al., 2017). However, recent 16S rRNA gene-based high-throughput sequencing surveys have revealed that rare taxa should also be considered as the indispensable component of full microbial communities due to their over-proportional roles in biogeochemical cycles and they could function as the “seed bank” of the vast gene pool (Sogin et al., 2006; Logares et al., 2015; Jousset et al., 2017). Thus abundant and rare taxa should be included in holistic analysis on microbial diversity.

Recent studies suggested abundant and rare biosphere might show contrasting response patterns to environmental and spatial factors when considering their relative importance in shaping community composition. For example, the abundant sub-community in freshwater lakes showed much stronger response to spatial factors than its rare counterpart (Liu et al., 2015; Yang et al., 2016). In contrast, abundant and rare taxa were shown to present similar biogeography in coastal saline lakes (Galand et al., 2009; Logares et al., 2013). Such inconsistency could be ascribed to different ranges of environmental factors (e.g., salinity) and geographic distance among those investigated ecosystems (Galand et al., 2009; Logares et al., 2013; Liu et al., 2015; Yang et al., 2016). However, few studies have ever differentiated abundant, and rare taxa from total microbial community when assessing microbial diversity and its response to environmental and spatial factors in hot springs.

The Tibetan Plateau (>4500 m above sea level) is located on the Indian-Eurasia collision orogenic belt, belonging to the Mediterranean-Himalayan hydrothermal areas (Wang et al., 2013). The southern Tibetan Plateau hosts one of the most active geothermal areas in the world and possesses a large number of hot springs with various environmental gradients, such as pH (3.0–8.6), temperature (30–97°C), and sulfate concentrations (3.3–850.5 mg/L) (Wang et al., 2013; Guo et al., 2014). So far several studies have been performed to investigate the microbial community in Tibetan hot springs. For example, clone library-based phylogenetic analysis showed that the bacterial communities were predominated by *Firmicutes, Proteobacteria, Cyanobacteria*, and *Chloroflexi* (Lau et al., 2009; Huang et al., 2011) and that archaeal and bacterial diversity did not show statistically significant correlation with temperature in the Tibetan hot springs (Lau et al., 2009; Huang et al., 2011). In contrast, another two 454 pyrosequencing-based studies uncovered a higher number of dominant phylogenetic groups and showed that temperature controlled the microbial structure in Tibetan hot springs (Song et al., 2013; Wang et al., 2013). However, in these previous microbial studies the rare sub-community was not differentiated from total microbial community due to the intrinsic limitations of clone library-based and/or 454 Pyrosequencing techniques (Jiang et al., 2018) and to the limited number of sampled hot springs (Song et al., 2013; Wang et al., 2013). Therefore, a holistic investigation is needed on the taxonomic diversity and potential ecological functions of total community, and abundant and rare sub-communities in Tibetan hot springs.

The aims of this study were to investigate (1) the taxonomic diversity and potential ecological functions of the microbial communities (in total, abundant, and rare biospheres) in the Tibetan hot springs; (2) the influences of environmental variables on the ecological functions of the microbial communities in total, abundant, and rare biospheres; and (3) the relative importance of environmental variables (e.g., temperature) and spatial factors to shaping of microbial communities in total, abundant, and rare biospheres in the Tibetan hot springs.

## Materials and Methods

### Field Measurements and Sample Collection

**FIGURE 1:**
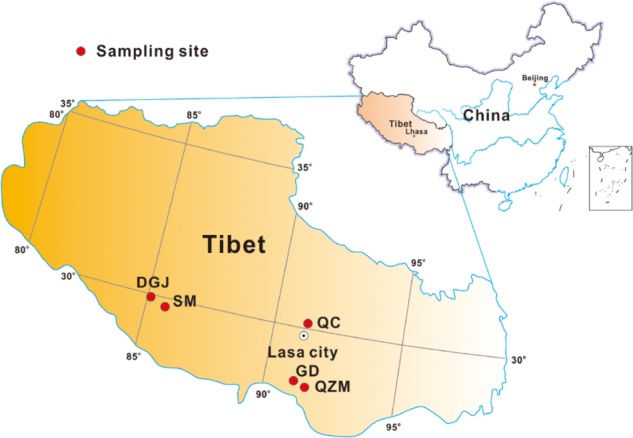
Geographic map showing the sampling locations in this study (including zones of QZM, DGJ, QC, SM, and GD as indicated in the M&M).

In summer of 2015 and 2016, field measurements and sample collection were performed in a total of 66 hot springs from five areas located on the southern Tibetan Plateau. These five sampling areas included the Quzemu zone (QZM) in Cuona County located in the valley areas from the Gonzáles Mountain to southern Tanggula Mountain, the Daggyai (DGJ) zone in Angren County, the Qucai (QC) zone in Naqu County, the Gudui (GD) zone in Cuomei County, and the Semi (SM) zone in Luozha County (**Figure 1**). Most hot springs distributed in these five zones were shaped as small (diameter < 1 m) wells (deep or shallow) with or without small outlets (**Supplementary Figures S1A,B,G–L**), while only several hot springs were shaped as lakes (**Supplementary Figure S1F**). Some hot spring waters flows directly out of the rock of the mountain in the QZM zone (**Supplementary Figures S1C,D**). Most hot springs in the DGJ zone were featured as geysers (**Supplementary Figures S1E–G**).

In the field, at each sampled hot spring water temperature, pH and concentrations of dissolved oxygen (DO) and Fe^2+^ were measured with a temperature/pH probe (DR850, HACH Company, CO, United States) and Hach kits. For water chemistry measurement, about 50 mL hot spring water was filtered through polycarbonate membrane filters (pore size 0.22 μm). A half of the resulting filtrate was collected into polyethylene bottles for major anion measurement, and the other half of resulting filtrate was collected for major cation measurement into glass bottles supplemented with concentrated HNO_3_ (to a final concentration of 0.1 M). For dissolved organic carbon (DOC) analysis, hot spring water was filtrated through 0.7-mm Whatman GF/F filters followed by acidification with concentrated phosphoric acid (to a final concentration of 0.1 M). The resulted DOC samples were stored on ice in the field and during transportation, and were then stored at 4°C in the laboratory until further analysis. After field measurements, the sediment/water interface (top 1 cm) was collected for DNA extractions and total organic carbon (TOC) measurements from each studied hot spring and then stored on dry ice in the field and during transportation. On arrival in laboratory, the frozen samples were transferred to a -80°C freezer until further analyses.

### Laboratory Geochemical Analyses

Major cation and anion concentrations (e.g., K^+^, Na^+^, Ca^2+^, Mg^2+^, SO_4_^2-^, and Cl^-^) of the collected hot spring waters were measured by using ion chromatography (Dionex DX-600, United States). Salinity was obtained by summing the concentrations of the eight abovementioned major ions. Water DOC and sediment TOC concentrations were measured on a multi N/C 2100S analyzer (Analytik Jena, Germany). Before sediment TOC analysis, carbonates in the sediment samples were removed by overnight acidification with 1 N HCl, and then the acidified sediments were washed to neutral pH, dried in an oven and ground into fine powder.

### DNA Extraction and Sequencing

DNA was extracted from 0.5 g surface sediment samples using the Fast DNA SPIN Kit for Soil according to the manufacturer’s instruction (MP Biomedical, Solon, OH, United States). The extracted DNA was amplified with a universal 16S rRNA gene primer set of barcoded 515F (5′-GTGYCAGCMGCCGCGGTA-3′)/806R (5′-GGACTACVSGGGTATCTAAT-3′), and the detailed PCR conditions were described in a previous study (Tamaki et al., 2011). Briefly, a unique 12 bp barcode sequence was added between the sequencing adapter and the forward primer to differentiate among samples. In order to minimize technical artifacts (Zhou et al., 2011) and ruling out contaminations from the laboratory reagents, triplicate PCRs and experimental blanks (replacement of DNA with the same amount of sterilized deionized water) were conducted for each sample. The successful PCR products were purified using a DNA Gel Extraction Kit (Axygen, Union City, CA, United States). The DNA quality and concentrations were assessed based on the absorbance ratios of 260/280 and 260/230 using a NanoDrop ND-1000 Spectrophotometer (NanoDrop Technology, DE, United States). The bar-coded amplicons (291 bp in length) from each sample were pooled with equimolar concentrations and then were sequenced by using an Illumina MiSeq platform (Caporaso et al., 2012).

### Data Processing and Statistical Analyses

In order to increase the validity of the OTU grouping and to make the classification of intermediate and rare taxa more credible in the sampled hot springs, the obtained Illumina sequencing data were filtered according to the strategies described previously (Lynch and Neufeld, 2015). The data analyses were following the UPARSE pipeline (Edgar, 2013). Briefly, the paired reads were joined using FLASH (fast length adjustment of short reads) with default settings (Magoè and Salzberg, 2011). Both forward and reverse primer sequences were removed from the resulting joined reads, which were then demultiplexed and quality filtered using QIIME v1.8.0 with *split*_*libraries*_*fastq*.py script (Caporaso et al., 2012). Reads having the following properties were discarded: (1) containing more than three consecutive low quality (Phred quality score <30) bases, (2) containing ambiguous base, and (3) comprising consecutive high quality bases less than 75% of the total read length. Chimera checking was performed using the UCHIME module with *de novo* method in USEARCH^1^ (Edgar et al., 2011). Subsequently, qualified reads were de-replicated and clustered using the USEARCH. All the qualified reads were truncated to identical length with 235 nt. All singletons and reads with length less than 235 nt were discarded, and operational taxonomic units (OTUs) were defined at the 97% cutoff by using the UPARSE-OTU algorithm (Edgar, 2013). The OTU representative sequences were then selected and their taxonomy was assigned using the ribosomal data base project (RDP) classifier algorithm at the 80% threshold (Wang et al., 2007) against the SILVA 123 database in the QIIME program. The OTU table was rarefied to equal sequence number (*n* = 22938) for each sample with 1000 replicates, and then the alpha diversity (i.e., Simpson, Shannon, Equitability, and Chao1) was calculated at the 97% cutoff by QIIME. The rarefied OTU table was used for downstream analyses unless otherwise specified. Abundant and rare OTUs were arbitrarily defined as the OTUs with relative abundance of >1 and <0.01% within one sample, respectively (Galand et al., 2009), the intermediate OTUs were arbitrarily defined as the OTUs with relative abundance between 0.01–1%. In order to identify potential microbial ecological functions in the studied hot springs, the obtained OTUs were compared against the FAPROTAX 1.1 database to predict potential metabolic functions of the microbial community (Louca et al., 2016). The spearman correlation was assessed between the dominant taxa (or the dominant metabolic phenotypes) and the measured environmental factors with the use of “vegan” and “ggplot” in the R package.

In order to identify sites with similar water geochemistry, the measured environmental variables were subjected to cluster analysis by employing the PAST software with the unweighted pair group method with arithmetic mean (UPGMA) of Euclidean distance (Yang et al., 2013). In order to compare microbial similarity among the samples, UPGMA cluster analysis was conducted for the total, abundant and rare biosphere of the studied 66 hot spring samples based on Bray-Curtis dissimilarities of at the 97% cutoff. Mantel test was performed to assess the correlation between the microbial community (of total, abundant, intermediate, and rare biosphere) and the measured environmental variables/geographic distance by using the PAST software. In order to evaluate the relative importance of environmental and spatial factors in shaping microbial community, aggregated boosted tree (ABT) analysis was performed to quantitatively evaluate the relative influence of individual environmental factors and geographic distance on the distribution of microbial community using R package “gbm” (De’Ath, 2007). Variance inflation factors (VIFs) were computed to check the presence of collinearities among the environmental variables using the function VIF in the “car” package. The environmental variables that had VIF >10 (which indicated evident collinearities with the other measured environmental variables) were removed from subsequent ABT analysis until all VIFs of the variables were <10 (De’Ath, 2007). The parameters for ABT analysis were set as follows: distribution = gaussian, n.trees = 10000, shrinkage = 0.001, bag.fraction = 0.5, train.fraction = 0.5, cv.folds = 10, interaction.depth = 3.

In order to identify the difference in microbial community compositions of various temperature, the 66 hot spring samples were classified into the moderate temperature (32–73°C) and high temperature (73–86°C) groups (abbreviated as MT and HT hereinafter, respectively) depending on the upper temperature limit for photosynthesis (about 73°C) (Rothschild and Mancinelli, 2001). Similarity analysis (one-way ANOSIM) was performed based on the Bray-Curtis dissimilarity at the 97% cutoff to test for any significant dissimilarity in community compositions of the total, abundant, intermediate, and rare biosphere between the MT and HT groups of, respectively.

### Nucleotide Sequence Accession Numbers

The sequences retrieved in this study were deposited at the Sequence Read Archive (SRA) in the National Center for Biotechnology Information (NCBI) under the BioProject accession no. SRP101393.

## Results

### Environmental Parameters of the Sampled Hot Springs

The hot springs in this study possessed a range of temperature (32–86°C), pH (3.0–9.5), salinity (0.13–1.32 g/L), and DOC (0–958.60 mg/L) and TOC (0–12.60%) contents (**Supplementary Table S1**). Most of the QZM, QC, SM, and GD hot springs had nearly neutral pH (6.5–8.0), while most of the DGJ hot springs were neutral to slightly alkaline (pH 7.0–9.5), except the acidic DGJ_17 (pH = 3.0), DGJ_18 (pH = 4.0), and SM_3 (pH = 3.4) hot springs (**Supplementary Table S1**). Geochemistry clustering analysis showed that the sampled hot springs were clustered on the basis of their locations, suggesting that hot springs from different zones were distinct with respect to geochemistry (**Supplementary Figure S2**). Comparatively, the DGJ hot springs exhibited lower contents of DOC, TOC, SO_4_^2-^, and Cl^-^ than those in other four sampling zones (**Supplementary Table S1**). In addition, most of the samples from one sampling location were clustered together according to their temperature (i.e., MT vs. HT) in the QZM and DGJ zones.

### Microbial Diversity and Composition of the Total, Abundant, Intermediate, and Rare Biosphere

**FIGURE 2:**
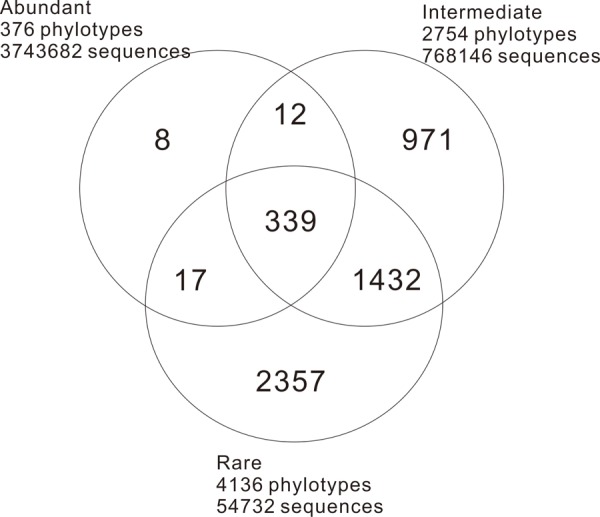
Venn diagram illustrating the distribution structure of the abundant, intermediate, and rare phylotypes (OTUs at the 97% cutoff) in the sampled hot springs.

**FIGURE 3:**
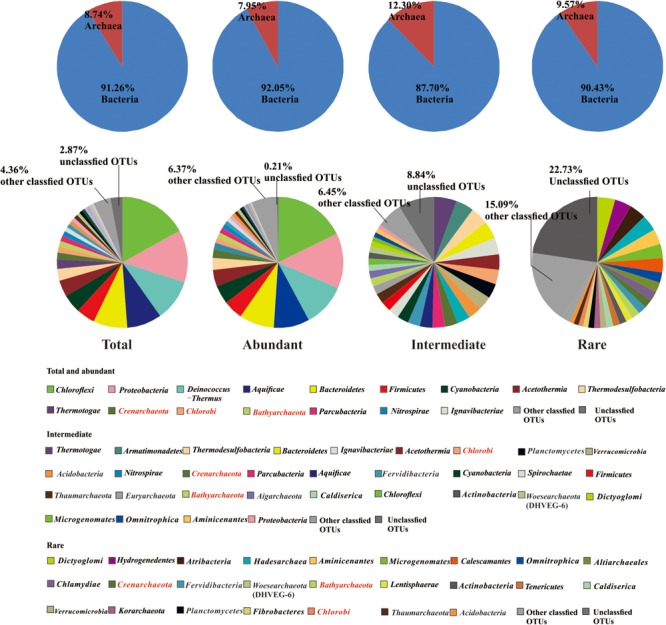
Relative abundance of bacteria and archaea (the upper row), and microbial community composition (at the phylum level) of the total, abundant, intermediate, and rare biospheres (the lower row) (in the caption, characters in red indicated the phyla occurring among the abundant, intermediate, and rare biospheres).

A total of 4566560 quality sequence reads were obtained from the 66 hot springs samples with an average of 69190 sequence reads per sample. The Goods coverage was 96–100%, indicating that the number of sequence reads was sufficient to capture most taxa in each sample (**Table 1**). The observed OTUs, and Shannon and Chao 1 indices ranged 65.11–516.85, 2.32–6.53, and 100.14–758.66 in the studied hot springs, respectively (**Table 1**). A total of 5136 OTUs were obtained in the total biosphere. A total of 376, 2754, and 4136 OTUs were identified as abundant, intermediate, and rare taxa, respectively. In total, 339 OTUs were common among the abundant, intermediate, and rare biosphere; 351 OTUs were common between the abundant and intermediate biospheres, 1762 OTUs were common between the intermediate and rare biospheres, and 356 OTUs were common between the abundant and rare biospheres (**Figure 2**). In addition, 8, 971 and 2357 OTUs were unique in the abundant, intermediate, and rare biospheres, respectively (**Figure 2**). These identified abundant, intermediate, and rare taxa covered 81.98, 16.82, and <2% of the total obtained quality sequence reads, respectively. Further phylogenic taxonomy analysis showed that about 91.26, 92.05, 87.70, and 90.43% of the obtained total, abundant, intermediate, and rare OTUs were affiliated with bacteria, and 8.74, 7.95, 12.30, and 9.57% of the total, abundant, intermediate, and rare OTUs were identified as archaea (**Figure 3**).

**Table 1:** Alpha diversity indices at the 97% OTU level of 16S rRNA gene libraries of the studied hot spring samples (re-sampling 22938 reads in each sample was performed for 1000 replicates).

Sample ID	No. of obtained sequence reads	Observed species	Goods coverage	Simpson	Shannon	Equitability	Chao1
QZM-1	99313	188.63	0.98	0.84	3.75	0.50	324.95
QZM-2	101178	278.46	0.98	0.89	4.51	0.56	489.47
QZM-3	109007	200.39	0.98	0.83	3.47	0.45	352.13
QZM-4	49444	286.03	0.98	0.84	4.45	0.55	440.87
QZM-5	52532	340.44	0.97	0.91	5.25	0.62	527.17
QZM-6	57095	286.18	0.98	0.93	4.93	0.60	463.18
QZM-7	109986	294.10	0.97	0.91	5.05	0.62	489.71
QZM-9	63729	361.93	0.97	0.87	5.10	0.60	541.96
QZM-10	51200	326.03	0.98	0.94	5.53	0.66	472.67
QZM-11	50457	297.66	0.98	0.92	5.04	0.61	443.44
QZM-12	57524	302.96	0.98	0.88	4.96	0.60	446.97
QZM-13	67777	305.12	0.98	0.92	5.14	0.62	471.55
QZM-14	73807	296.95	0.98	0.93	5.07	0.62	447.69
QZM-15	117181	209.36	0.98	0.88	4.11	0.53	368.35
QZM-16	182398	190.90	0.98	0.55	2.32	0.31	359.79
QZM_17	29163	165.13	0.99	0.95	5.20	0.71	219.03
QZM_18	56895	516.85	0.96	0.96	6.53	0.72	758.66
QZM_19	26561	268.23	0.98	0.90	5.11	0.63	349.55
QZM_20	43788	231.58	0.98	0.91	4.70	0.60	331.58
QZM_21	62016	176.90	0.99	0.94	5.07	0.68	247.80
QZM_22	79462	298.12	0.98	0.94	5.61	0.68	405.51
QZM_23	70841	138.71	0.99	0.91	4.28	0.60	221.68
QZM_24	46399	99.06	0.99	0.89	4.04	0.61	147.17
DGJ-1	132844	251.24	0.98	0.91	4.65	0.58	423.53
DGJ-2	143530	219.09	0.98	0.77	3.38	0.43	397.54
DGJ-3	114267	271.51	0.98	0.68	3.59	0.44	475.56
DGJ-4	130429	225.44	0.98	0.78	3.70	0.47	383.50
DGJ-5	122686	283.36	0.98	0.86	4.46	0.55	463.98
DGJ-6	68683	315.41	0.98	0.87	4.68	0.56	484.45
DGJ-8	108040	200.73	0.98	0.85	3.68	0.48	354.82
DGJ-9	157980	193.38	0.98	0.73	2.92	0.38	354.23
DGJ-10	135547	189.06	0.98	0.78	3.35	0.44	344.29
DGJ-11	75643	294.15	0.98	0.90	4.75	0.58	480.93
DGJ-12	101400	234.22	0.98	0.86	4.02	0.51	386.64
DGJ-13	29279	162.03	0.99	0.85	3.56	0.49	241.14
DGJ-14	29294	352.43	0.98	0.94	5.75	0.68	508.37
DGJ-15	135450	237.17	0.98	0.70	3.39	0.43	428.67
DGJ-16	56534	385.42	0.97	0.94	5.65	0.66	587.64
DGJ_17	56762	102.70	0.99	0.87	3.90	0.58	140.52
DGJ_18	53381	113.17	0.99	0.88	3.73	0.55	164.94
DGJ_19	61567	173.37	0.99	0.81	3.82	0.51	285.08
DGJ_20	46615	114.25	0.99	0.91	4.03	0.59	184.79
DGJ_21	76521	208.24	0.99	0.90	4.75	0.62	308.89
DGJ_22	57436	88.71	1.00	0.88	3.91	0.60	109.95
DGJ_23	44419	245.29	0.98	0.94	5.42	0.68	352.14
DGJ_24	58987	112.92	0.99	0.85	3.57	0.52	181.97
DGJ_25	62974	65.78	1.00	0.84	3.47	0.57	100.14
DGJ_26	88622	78.31	1.00	0.84	3.55	0.57	115.61
DGJ_27	57592	108.92	0.99	0.86	3.75	0.55	146.63
DGJ_28	32667	102.34	0.99	0.79	3.22	0.48	154.84
DGJ_29	47858	363.72	0.97	0.96	5.94	0.70	527.37
DGJ_30	51493	100.60	0.99	0.90	3.91	0.59	171.92
QC01	46085	104.13	0.99	0.88	3.94	0.59	139.85
QC02	42925	247.94	0.98	0.90	4.65	0.58	350.54
QC03	34831	249.25	0.98	0.93	5.01	0.63	353.16
QC04	32549	193.66	0.99	0.92	4.84	0.64	252.14
QC05	36372	420.48	0.97	0.94	5.56	0.64	639.77
SM_1	60261	115.12	0.99	0.88	4.06	0.59	166.33
SM_2	55048	111.93	0.99	0.89	4.08	0.60	175.46
SM_3	42195	65.11	0.99	0.78	2.91	0.48	107.86
SM_4	52521	116.61	0.99	0.86	3.84	0.56	156.92
GD_1	32301	104.89	0.99	0.79	2.96	0.44	158.13
GD_2	28471	183.07	0.99	0.92	4.62	0.62	249.24
GD_3	22938	300.01	0.98	0.96	5.79	0.70	394.81
GD_4	50119	285.79	0.98	0.92	4.99	0.61	454.78
GD_5	33691	149.03	0.99	0.77	3.37	0.47	239.37

The abundant sub-community and total community were composed of similar dominant phyla and classes: the top 10 most dominant phyla of the abundant sub-community were *Chloroflexi, Proteobacteria, Deinococcus-Thermus, Aquificae, Bacteroidetes, Firmicutes, Cyanobacteria, Acetothermia, Thermodesulfobacteria*, and *Thermotogae* (**Figure 3** and **Supplementary Table S2**), and the top 10 most dominant classes were *Deinococci, Chloroflexia, Aquificae, Betaproteobacteria, Sphingobacteriia, Cyanobacteria, Gammaproteobacteria, Bacilli, Anaerolineae*, and *Thermodesulfobacteria* (**Figure 3** and **Supplementary Table S3**). In comparison, the phylogenetic composition of the intermediate sub-community showed little difference from the abundant sub-community. Specifically, the top 10 most dominant phyla in the intermediate sub-community were *Thermotogae, Armatimonadetes, Thermodesulfobacteria, Bacteroidetes, Ignavibacteriae, Acetothermia, Chlorobi, Planctomycetes, Verrucomicrobia*, and *Acidobacteria* (**Figure 3** and **Supplementary Table S2**), with *Thermotogae, Thermodesulfobacteria, Gammaproteobacteria, Ignavibacteria, Solibacteres, Alphaproteobacteria, Chlorobia, Sphingobacteriia, Planctomycetacia*, and *Nitrospira* being the top 10 most dominant Classes (**Supplementary Table S3**). In contrast, the rare sub-community showed apparently different phylogenetic composition from its abundant counterpart: the top 10 most dominant phyla in the rare biosphere were *Dictyoglomi, Hydrogenedentes, Atribacteria, Hadesarchaea, Aminicenantes, Microgenomates, Calescamantes, Omnitrophica, Altiarchaeales*, and *Chlamydiae*. Correspondingly, with *Acidimicrobiia, Opitutae, Coriobacteriia, Dictyoglomia, Fimbriimonadia, Chthonomonadetes, Thermomicrobia, Caldilineae, Verrucomicrobia*, and *Thermoleophilia* being dominant Classes (**Supplementary Table S2**).

Further analysis showed that all the detected dominant phyla in the abundant biosphere or most (44.09%) of the detected dominant phyla in the rare biosphere can be observed in the intermediate biosphere. Meanwhile, some phyla (e.g., *Crenarchaeota, Bathyarchaeota*, and *Chlorobi*) were common among the abundant, intermediate, and rare biospheres (**Figure 3** and **Supplementary Table S2**). The total and abundant biospheres showed similar community structures among the studied Tibetan hot springs, while they were different from the intermediate and rare biosphere (**Supplementary Figures S3A–D**). The HT samples tended to be clustered together for the total microbial communities, and abundant and rare sub-communities. In addition, the total and rare communities exhibited evident biogeographic patterns, in contrast with no biogeographic pattern in the intermediate and abundant biosphere (**Supplementary Figures S3A–D**). Furthermore, one-way ANOSIM test indicated that the community structures of the total, abundant and intermediate biospheres showed more significant (*p* < 0.01 vs. *p* < 0.05) difference between the MT and HT hot springs than that of the rare biosphere did (**Table 2** and **Supplementary Tables S4–S7**).

**Table 2:** One-way ANOSIM test on the difference in community structures of the total, abundant, intermediate, and rare biospheres between the MT and HT hot springs.

Biosphere	OTUs	R	P
Total	MT vs. HT	0.193	<0.001
Abundant	MT vs. HT	0.105	<0.01
Intermediate	MT vs. HT	0.211	<0.001
Rare	MT vs. HT	0.094	<0.05

### Influence of Environmental and Spatial Factors on the Microbial Distribution

The Mantel test showed that both environmental (temperature, pH, Fe^2+^, salinity, DOC, and TOC) and spatial factors (GD, geographical distance) were significantly (*p* < 0.05) correlated with the total communities and the abundant, intermediate, and rare sub-communities (**Table 3**). Specifically, the total community was significantly (*p* < 0.05) correlated with temperature, salinity, DOC, GD, pH, Fe^2+^, and TOC (**Table 3**); the abundant sub-community was significantly correlated with temperature, GD, pH and salinity (**Table 3**). While the intermediate and rare sub-communities were significantly (*p* < 0.05) correlated with DOC, salinity and GD (**Table 3**). In addition, the common phyla (i.e., *Crenarchaeota, Chlorobi*, and *Bathyarchaeota*) of the abundant and rare biospheres showed different responses to the measured environmental factors (**Table 4**). For example, *Crenarchaeota* was only significantly correlated with DOC (*p* < 0.01), and *Chlorobi* was significantly correlated with salinity (*p* < 0.001), TOC (*p* < 0.01), pH (*p* < 0.01), and DOC (*p* < 0.05). While *Bathyarchaeota* showed no correlations with the measured environmental factors. In contrast, these three common phyla were significantly correlated with the graphical distance (*p* < 0.01) (**Table 4**).

**Table 3:** Mantel test showing correlation between the individual measured environmental parameters and the microbial communities (in the total, abundant, intermediate, and rare biospheres) of the sampled hot springs.

Physicochemical parameters	Total OTUs		Abundant OTUs		Intermediate OTUs		Rare OTUs	
	*R*	*P*	*R*	*P*	*R*	*P*	*R*	*P*
T	0.312	<0.001	0.320	<0.001	0.163	<0.05	0.019	0.600
pH	0.156	<0.05	0.149	<0.05	0.140	<0.05	0.066	0.223
SAL	0.225	<0.001	0.171	<0.01	0.236	<0.001	0.146	<0.05
Fe2	0.155	<0.05	0.034	0.206	–0.002	0.498	–0.045	0.805
DOC	0.306	<0.001	0.049	0.120	0.188	<0.001	0.282	<0.001
TOC	0.110	<0.05	0.739	0.059	0.117	<0.05	0.052	0.167
GD	0.159	<0.001	0.133	<0.001	0.137	<0.001	0.049	<0.05

*P-value showing significant correlations (*P* < 0.05) were highlighted in gray. SAL, salinity; DOC, dissolved organic carbon; TOC, total organic carbon; GD, geographic distance; Fe2, Fe^2+^.*

**Table 4:** Mantel test showing correlation between individual environmental parameters and phyla of *Crenarchaeota, Chlorobi*, and *Bathyarchaeota* common in the abundant, intermediate, and rare biospheres of the sampled hot springs.

Physicochemical parameters	*Crenarchaeota*		*Chlorobi*		*Bathyarchaeota*	
	*R*	*P*	*R*	*P*	*R*	*P*
T	0.083	0.053	0.100	0.029	–0.046	0.801
pH	0.013	0.394	0.191	<0.01	–0.030	0.644
SAL	0.079	0.081	0.297	<0.001	0.056	0.182
Fe2	–0.035	0.082	–0.033	0.797	–0.035	0.785
DOC	0.113	<0.01	0.088	<0.05	–0.001	0.472
TOC	0.043	0.148	0.135	<0.01	–0.032	0.740
GD	0.069	<0.01	0.101	<0.01	0.845	<0.01

*P-value in gray background indicated a significant correlation (*p* < 0.05). SAL, salinity; DOC, dissolved organic carbon in hot spring water; TOC, total organic carbon in hot spring sediment; GD, geographic distance; Fe2, Fe^2+^.*

**FIGURE 4:**
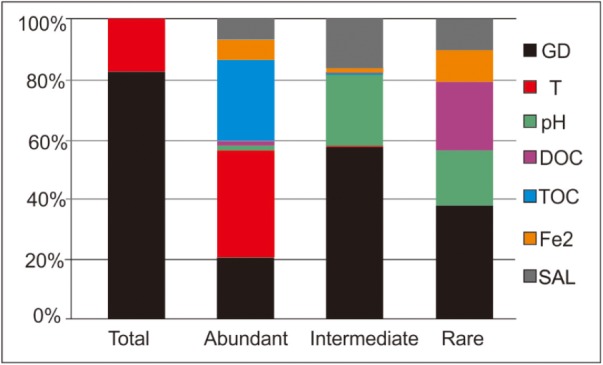
ABT analysis showing the relative influence of individual environmental factors and geographic distance on shaping of microbial communities of the total, abundant, intermediate, and rare biospheres (SAL, salinity; DOC, dissolved organic carbon; TOC, total organic carbon; GD, geographic distance; Fe2, Fe^2+^).

The ABT analysis showed that the relative importance of the measured variables differed with respect to their contribution to shaping of the microbial compositions of the studied hot springs (**Table 3** and **Figure 4**). For shaping of the total community, spatial factors (GD) exhibited higher contribution (83.4 vs. 16.5%) than temperature, while other environmental factors (such as TOC, DOC, Fe^2+^, and salinity) show no contribution; for shaping of the intermediate biosphere, spatial factors (GD) also exhibited higher contribution (56.8 vs. 43.2%) than the measured environmental factors. In contrast for shaping of the abundant and rare sub-communities, the environmental factors exhibited higher contributions (79.2 vs. 20.8% and 61.5 vs. 38.5%, respectively) than the spatial factors (**Figure 4**). For the abundant sub-community, the top three influencing factors were temperature, TOC and graphical distance, each contributing 36.2, 26.7, and 20.8% of the observed microbial variation, respectively. In contrast, for the intermediate sub-community, the top three influencing factor were spatial factors, pH and salinity, each contributing 56.8, 23.5, and 16.5% of the observed microbial variation, while temperature showed only 0.2% of contribution to shaping of the intermediate sub-community. Similarly, for the rare sub-community, the top three influencing factor were spatial factors, DOC content and pH, each contributing 38.5, 22.5, and 18.6% of microbial variation, respectively. However, temperature showed no observed contribution to shaping of the rare sub-community (**Figure 4**).

### Correlations Between the Dominant Taxa of the Abundant, Intermediate, and Rare Biospheres and the Environmental Factors

**FIGURE 5:**
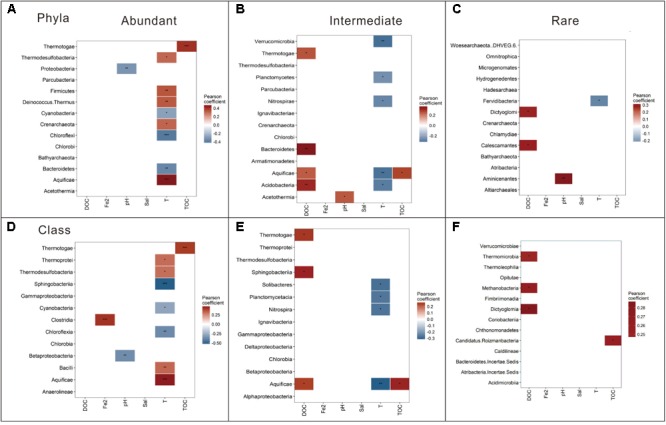
Pearson correlation between the relative abundance of the dominant taxa (at the phylum and class levels) and the measured environmental variables for the abundant **(A,D)**, intermediate **(B,E)**, and rare **(C,F)** biospheres. ^∗∗∗^*p <* 0.001; ^∗∗^*p <* 0.01; and ^∗^*p <* 0.05; Sal, salinity; DOC, dissolved organic carbon; TOC, total organic carbon; Fe2, Fe^2+^.

Spearman correlation analysis revealed that the relative abundance of dominant taxa (at the phylum and class levels) were correlated with different environmental factors. The dominant taxa in the abundant biosphere were significantly correlated with temperature; the dominant taxa in the intermediate biosphere were significantly correlated with DOC and temperature; while the dominant taxa in the rare biosphere were significantly correlated with DOC (**Figure 5**). Specifically, different phyla and classes of one biosphere (abundant, intermediate or rare) exhibited diverse correlations with the measured environmental factors. Take the abundant biosphere for an example: the relative abundance of the phyla (and their corresponding Classes) such as *Aquificae* (*Aquificae*), *Deinococcus-Thermus, Firmicutes* (*Bacilli*), *Thermodesulfobacteria* (*Thermodesulfobacteria*), and *Crenarchaeota* (*Thermoprotei*) showed positive correlations (*p* < 0.05) with temperature. Conversely, relative abundance of *Chloroflexi* (*Chloroflexia*), *Bacteroidetes* (*Sphingobacteriia*), and *Cyanobacteria* (*Cyanobacteria*) showed negative correlations (*p* < 0.05) with temperature. In addition to these abovementioned major phyla or class in the abundant biosphere showing significant response to temperature, some phyla were significantly correlated with environmental variables rather than temperature. For example, *Thermotogae* (*Thermotogae*) and *Proteobacteria* (*Betaproteobacteria*) showed positive and negative correlations with TOC content (*p* < 0.001) and pH (*p* < 0.05), respectively.

Moreover, the common phyla (and their corresponding Classes) of the abundant and intermediate biospheres exhibited different responses to environmental factors. For example, *Aquificae* (*Aquificae*), *Bacteroidetes* (*Sphingobacteriia*), and *Thermotogae* (*Thermotogae*) in the intermediate biosphere exhibited positive correlations (*p* < 0.05) with DOC content, in contrast with their significant correlations (*p* < 0.01) with temperature in the abundant biosphere. Besides, the dominant phyla of the intermediate biosphere (and their corresponding classes) such as *Planctomycetes* (*Planctomycetacia*), *Verrucomicrobia, Nitrospirae* (*Nitrospira*), *Acidobacteria* (*Solibacteres*), and *Aquificae* (*Aquificae*) exhibited negative correlations (*p* < 0.05) with temperature (**Figure 5**). In contrast with the total, abundant and intermediate biospheres, only a few major phyla or classes in the rare biosphere exhibited correlations with the measured environmental factors. For example, *Aminicenantes* showed a significant positive correlation (*p* < 0.01) with pH; *Dictyoglomi* (*Dictyoglomia*) and *Calescamantes* showed a positive correlation with DOC content; and *Fervidibacteria* showed a positive correlation with temperature (**Figure 5**).

### Potential Microbial Ecological Functions of the Total, Abundant, Intermediate, and Rare Communities and Their Correlation With Environmental Factors

**FIGURE 6:**
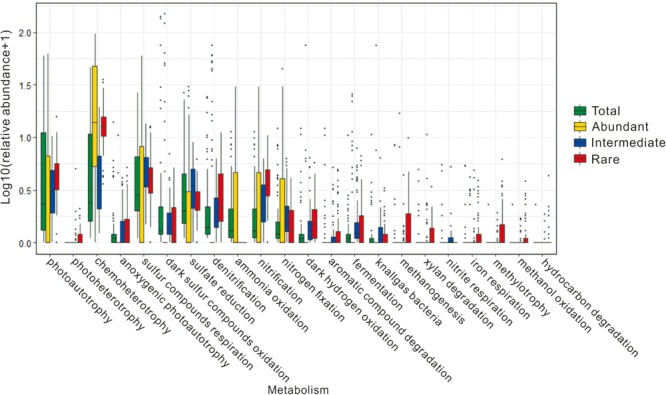
The potential microbial ecological functions identified from prediction and their relative abundance in the total, abundant, intermediate, and rare biospheres of the studied hot springs.

About 1.87–62.65, 24.60–98.60, 3.83–86.48, and 67.97–93.34% of the obtained OTUs could be predicted with potential microbial ecological functions in the total, abundant, intermediate, and rare biospheres, respectively. In general, the predicted microbial ecological functions were related to carbon, sulfur and nitrogen cycles in these studied hot springs (**Figure 6** and **Supplementary Table S8**). In the total biosphere, twenty-two microbial ecological functions (relative abundance >0.01%) were identified, including chemoheterotrophy, dark sulfur oxidation, photoautotrophy, sulfur respiration, sulfate reduction, denitrification, ammonia oxidation, nitrification, nitrogen fixation, anoxygenic photoautotrophy, dark hydrogen oxidation, aromatic compound degradation, fermentation, knallgas bacteria, methanogenesis, xylan degradation, nitrite respiration, iron respiration, photoheterotrophy, methylotrophy, methanol oxidation, and hydrocarbon degradation. Fourteen of these predicted microbial functions were common among the abundant, intermediate, and rare biospheres, including chemoheterotrophy, dark sulfur oxidation, photoautotrophy, sulfur respiration, sulfate reduction, denitrification, nitrification, nitrogen fixation, anoxygenic photoautotrophy, dark hydrogen oxidation, fermentation, knallgas bacteria, methanogenesis, and iron respiration. In contrast, some of the predicted ecological functions were only present in the abundant, intermediate, or rare biosphere. For example, ammonia oxidation was only observed in the abundant biosphere, while photoheterotrophy, aromatic compound degradation, xylan degradation, methylotrophy, methanol oxidation, and hydrocarbon degradation were only found in the intermediate and rare biospheres (**Figure 6** and **Supplementary Table S8**).

**FIGURE 7:**
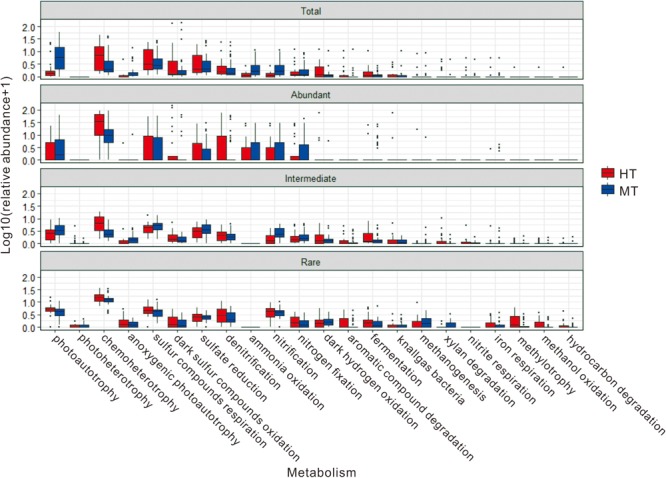
Relative abundance of the predicted ecological functions in total, abundant, intermediate, and rare biospheres of the MT and HT hot springs.

In addition, some microbial ecological functions identified from the total, abundant and intermediate biospheres exhibited different relative abundance between the MT and HT hot springs (**Figure 7** and **Supplementary Table S9**). For example, in the total and abundant biospheres, the relative abundance of photoautotrophy was higher (8.85 vs. 2.33% in the total biosphere and 7.09 vs. 3.88% in the abundant biosphere) in the MT hot springs than their counterparts in the HT hot springs, while the relative abundance of chemoheterotrophy (4.95 vs. 10.75% in the total biosphere and 19.46 vs. 33.82% in the abundant biosphere) and dark oxidation of sulfur compounds (6.21 vs. 8.11% in the total biosphere and 5.11 vs. 9.90% in the abundant biosphere) were lower in the MT hot springs than their counterparts in the HT hot springs. For the intermediate and rare biosphere, however, the identified microbial ecological functions varied little in their relative abundance between the studied MT and HT hot springs (**Figure 7** and **Supplementary Table S9**).

**FIGURE 8:**
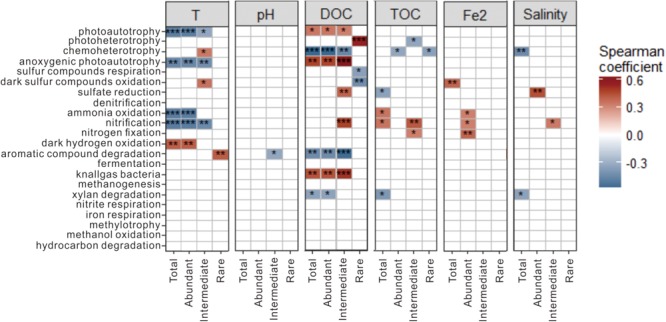
Spearman correlations between the relative abundance of the identified microbial ecological functions and the measured environmental variables for the total, abundant, intermediate, and rare biospheres. ^∗∗∗^*p <* 0.001; ^∗∗^*p <* 0.01; and ^∗^*p <* 0.05.

Spearman correlation analysis showed that temperature, DOC and TOC were the key factors influencing some of the predicted potential microbial ecological functions (relative abundance >1%) in the total, abundant and intermediate biospheres (**Figure 8**). For example, photoautotrophy showed negative and positive correlations with temperature (*p* < 0.001) and DOC (*p* < 0.05), respectively; ammonia oxidation and nitrification showed negative and positive correlations with temperature (*p* < 0.001) and TOC (*p* < 0.05), respectively; anoxygenic photoautotrophy showed negative and positive correlations with temperature (*p* < 0.01) and DOC (*p* < 0.01), respectively; and chemoheterotrophy showed a negative correlation with DOC (*p* < 0.01). In contrast, some dominant microbial ecological functions in the rare biosphere were significantly correlated with DOC content (**Figure 8**). For example, photoheterotrophy (*p* < 0.001) and sulfur respiration/dark sulfur oxidation (*p* < 0.05) showed positive and negative correlations with DOC content, respectively.

## Discussion

### Discriminating Between the Abundant and Rare Sub-Communities Availed Better Understanding of the Microbial Structure in Tibetan Hot Springs

It is notable that differentiating the abundant and rare sub-communities from the total biosphere will improve our understanding of the microbial structure in the studied hot springs. The abundant sub-community in this study showed similar composition (at the phylum level as shown in **Figure 3** and **Supplementary Table S2**) to the total microbial community reported previously in neutral hot springs of China (Tibetan and Yunnan) and Malaysia (Song et al., 2013; Wang et al., 2013; Chan et al., 2017). However, the rare taxa were not differentiated from total biosphere in those previous studies. In contrast, rare taxa were separated from their abundant counterpart in this study, and most of them (at the phylum level as shown in **Figure 3** and **Supplementary Table S2**) belonged to either newly proposed phyla and/or candidate divisions, which were commonly detected at a lower relative abundance in the hot springs of the United States (Yellowstone National Park and California), Japan, and India (Hedlund et al., 2014; Badhai et al., 2015). So it is necessary to separate rare sub-communities from their abundant counterpart when assessing microbial diversity and community structures in hot springs. In addition, the bacterial diversity identified in this study was higher (49 vs. 42 phyla) than our previous study (Wang et al., 2013). Such difference could be ascribed to the intrinsic limitations of the sequencing techniques (Illumina sequencing vs. 454 Pyrosequencing between this and our previous study) (Jiang et al., 2018).

In addition, the abundant, intermediate, and rare status of some microbial taxa might transform to each other when environmental conditions or spatial factors differ. For example, in this study, the phyla of *Crenarchaeota* (belonging to Class of *Desulfurococcales, Thermoproteales, Sulfolobales*, and *Fervidicoccales*), *Bathyarchaeota*, and *Chlorobi* (belonging to class of *Chlorobiales*) were common among the abundant, intermediate, and rare sub-communities, but they showed different responses to environmental and spatial factors (**Table 4**). Besides, the rare phyla of *Dictyoglomi, Armatimonadetes*, and *Thaumarchaeota* identified in this study were ever detected as abundant phyla in previous researches on Tibetan hot springs (Song et al., 2013; Wang et al., 2013, 2014), such finding could be ascribed to the fact that the rare sub-community can serve as part of the seed bank and contribute to ecosystem dynamics and even become dominant under favorable conditions (Galand et al., 2009; Brazelton et al., 2010; Hugoni et al., 2013). Moreover, the common phyla of *Crenarchaeota, Bathyarchaeota*, and *Chlorobi* differed in their phylogenetic compositions between the abundant and rare sub-communities (e.g., the *Thermofilum* genus-like vs. unclassified OTUs in *Thermoproteales* between the rare and abundant sub-communities). These findings suggested that abundant and rare taxa may occupy different ecological niches and they together maintain ecological relevance in hot springs (Jousset et al., 2017). Note that the observed abundant, intermediate, and rare status transformation of some microbial taxa may be caused by sampling artifacts (e.g., random sampling) (Zhou et al., 2008) and intrinsic limitations of high-throughput sequencing techniques (Jiang et al., 2018), which could be improved by employing technical and biological replicates (Zhou et al., 2011). Due to lack of biological replicates, the rare biosphere diversity of the studied hot springs may be overestimated in this study. However, the current data in the present study could at least give a sketch of rare biosphere diversity in the studied Tibetan hot springs.

### Different Influences of Environmental and Spatial Factors on the Total, Abundant and Rare Sub-Communities

It is noteworthy that total microbial community, abundant, intermediate, and rare sub-communities responded differently to environmental and geographical factors in the studied Tibetan hot springs. Previous microbial research in hot springs did not differentiate the abundant, intermediate, and rare taxa from the total microbial community, and temperature was shown to be the key factor in shaping microbial structures in hot springs (Miller et al., 2009; Cole et al., 2013; Wang et al., 2013; Chan et al., 2017; Saxena et al., 2017). In contrast with this study, temperature was only found as the most important environmental factor affecting the abundant sub-community, whereas it was not among the most significant factors influencing the intermediate and rare sub-communities (**Figure 4**). In addition to temperature, organic substrates were shown to shape microbial diversity in other previous research, which did not discriminate abundant and rare sub-communities (Menzel et al., 2015; Chan et al., 2017). Such different responses of abundant and rare sub-communities to environmental variables suggested that different environmental factors could account for the distribution of various microbial taxa. In addition, the rare taxa contributed the majority of the microbial diversity (Galand et al., 2009; Pedrós-Alió, 2012). So it is reasonable to observe that different environmental factors shaped community structures of total, abundant and rare biospheres of the studied Tibetan hot springs, which was also in accordance with previous findings in oceans (Pedrós-Alió, 2012; Logares et al., 2014).

Moreover, the dominant taxa (at the phylum and class levels) in the abundant, intermediate, and rare sub-communities also responded differently to the measured environmental factors. For example, most phyla (class) of the abundant biosphere exhibited positive or negative correlations with temperature (as shown in **Figure 5**), which was consistent with previous findings for total microbial community in hot springs (Cole et al., 2013; Hou et al., 2013; Wang et al., 2013). In contrast, some phyla (class) in the abundant sub-community showed significant correlation with non-temperature environmental factors (For example, *Thermotoga* and *Proteobacteria* were significantly correlated with TOC content and pH, respectively). In addition, some common phyla between the abundant and intermediate sub-communities exhibited different responses to the measured environmental factors (For example, *Aquificae* in the intermediate sub-community exhibited a negative correlation with temperature and a positive correlation with DOC, while the *Aquificae* showed a positive correlation with temperature in the abundant sub-community). Such inconsistency could be ascribed to the fact that the common phyla between different sub-communities were composed of different taxa (**Supplementary Table S2**) and they might be characteristic of different growth and activity, as manifested in their relative abundance of the abundant (>1%), intermediate (0.01–1%) and rare (<0.01%) sub-communities.

It is also notable that spatial factors (e.g., GD) showed more contribution to shaping of the intermediate and rare sub-communities than to the abundant sub-community (**Figure 4**). This finding could be explained by the fact that the dispersal of rare taxa was more likely restricted among different ecosystems than that of their abundant counterparts (Logue, 2010; Liu et al., 2015). Furthermore, other unmeasured factors such as sediment mineralogy (Hou et al., 2013) and total nitrogen (Chan et al., 2017) might contribute to shaping of community structures of total, abundant and rare biospheres, which awaits further investigation.

### Diverse Microbial Metabolic Phenotypes and Their Correlations With Environmental Factors in the Abundant, Intermediate, and Rare Biospheres

It is not surprising to observe more potential microbial ecological functions (20 and 21 vs. 15) (**Supplementary Table S6**) predicted in the intermediate and rare biospheres than that in the abundant biosphere because the former contained more diverse microbial taxa (OTUs) (Lynch and Neufeld, 2015). This finding may suggested that the intermediate and rare sub-communities in hot springs played important roles in the ecological function cache and thus ensured the ecosystem stability in response to environmental change (Ashley et al., 2012; Lynch and Neufeld, 2015). In addition, the intermediate and rare sub-communities contained some unique microbial functions related to organic matter degradation such as methylotrophy, methanol oxidation, aromatic compound degradation, xylan degradation, and hydrocarbon degradation (**Figure 6** and **Supplementary Table S8**), whereas such microbial functions were not identified in the abundant sub-community. Such contrasting results suggested that the intermediate and rare sub-communities in the studied hot springs may play more important roles than their abundant counterparts in organic matter degradation. In contrast, the abundant sub-community also contained unique microbial functions (e.g., ammonia oxidation) that were not identified in the intermediate and rare sub-communities. Such distinct distribution of microbial ecological functions between the abundant and rare sub-communities suggested that the abundant and rare taxa might occupy different ecological niches in hot spring ecosystems. However, further research is required to figure out the roles that the abundant and rare taxa each play in the ecological functions of the studied hot springs.

In addition to the abovementioned different ecological function compositions among the abundant, intermediate, and rare biospheres, it is also interesting to observe that some microbial functions shift in their abundance between the total/abundant biospheres of the MT and HT hot springs. For example, photoautotrophy was the main carbon fixation pathway in the MT hot springs (8.85 and 6.24% in relative abundance in total and abundant biosphere, respectively), while lower relative abundance was detected for photoautotrophy in the HT hot springs (2.33% the total biosphere and 4.02% in the abundant biosphere). Such abundance difference could be ascribed to the temperature limitation on photosynthesis (Boyd et al., 2009; De Leon et al., 2013); ammonia oxidation and nitrification were dominant in the MT hot springs but became minor in the HT hot springs, which was consistent with findings for microbial ammonia oxidation and nitrification in previous metagenomic studies (Reigstad et al., 2008; Zhang et al., 2008; Jiang et al., 2010). In contrast, chemoheterotrophy and chemolithotrophy pathways (e.g., dark oxidation of sulfur compounds, respiration of sulfur compounds, sulfate reduction, and denitrification) were minor in the MT hot springs but became dominant in the HT hot springs. So the observed abundance shift of microbial functions between MT and HT hot springs suggested that temperature could be an important factor influencing the distributions of microbial ecological functions in the studied hot springs. In the meanwhile, other environmental factors (e.g., DOC and TOC) may also influence the distribution of the microbial ecological functions identified in this study. These findings were consistent previous metagenomic results (Badhai et al., 2015; Saxena et al., 2017).

Moreover, the predicted microbial functions in the abundant and rare sub-communities responded differently to the measured environmental variables. For examples, the relative abundance of photoautotrophy, anoxygenic photoautotrophy and nitrification was significantly correlated with temperature for the total, abundant and intermediate biospheres, but showed no correlation with temperature for the rare biosphere. In contrast, some of the identified microbial functions (e.g., photoheterotrophy, sulfur respiration, dark sulfur oxidation, and aromatic compound degradation) showed significant correlations with temperature and DOC for the rare biosphere, which however, was not observed for the total and abundant biospheres (**Figure 8**). Such different responses suggested that the potential ecological functions of abundant, intermediate, and rare taxa may differ among the hot springs of different environmental conditions (e.g., temperature, DOC, and TOC). Note that the obtained knowledge in this study may be limited about the environmental influence on microbial metabolic functions in the studied hot springs due to a large proportion of unclassified OTUs (**Figure 3**), a large proportion of OTUs without predicted ecological functions, and some unmeasured environmental variables, which awaits further investigation.

## Conclusion

The abundant and rare sub-communities possessed different phylogenetic compositions and potential ecological functions, and they responded differently to environmental and spatial factors in the studied Tibetan hot springs. Temperature was the most important factor shaping the community compositions of the total and abundant biospheres, but it only showed little influence on the intermediate and rare sub-communities, suggesting that the abundant, intermediate, and rare biosphere may be sensitive to different environmental factors. Spatial factor (GD) contributed more influence to the variation of the rare sub-community than that of the abundant sub-community, indicating abundant taxa were more easily dispersed than rare taxa among different hot springs. The predicted microbial ecological functions of the abundant and rare sub-communities responded differently to the measured environmental factors, suggesting that they may occupy different ecological niches in hot spring ecosystems. In addition, the intermediate and rare taxa may play important roles in organic degradation than their abundant counterparts in the Tibetan hot springs.

## Author Contributions

YZ, GW, and HJ conceived and designed the experiments. YZ, GW, JY, and WS collected samples and performed the experiments. YZ, JY, WL, and HJ analyzed the data. All of the authors assisted in writing the manuscript, discussed the results, and commented on the manuscript.

## Conflict of Interest Statement

The authors declare that the research was conducted in the absence of any commercial or financial relationships that could be construed as a potential conflict of interest.

## Footnotes

1 http://www.drive5.com/usearch/
