# Supplementary material for: Abundant and Rare Microbial Biospheres Respond Differently to Environmental and Spatial Factors in Tibetan Hot Springs
Source: Front Microbiol. 2018 Sep 19;9:2096. doi: 10.3389/fmicb.2018.02096 (PMC6156277; doi:10.3389/fmicb.2018.02096)

Table S1. Geographic and geochemical parameters of the studied hot springs on the Tibetan Plateau

Sampling Zone	Sample ID	Latitude	Longitude	Elevation (meters about sea level)	Temp. (^{0}C)	pH	DOC (mg/L)	TOC (%)	F (mg/L)	Cl (mg/L)	SO4 (mg/L)	Br (mg/L)	Na (mg/L)	K (mg/L)	Mg (mg/L)	Ca (mg/L)	Salinity (g/L)	Fe2 (mg/L)
QZM zone	QZM_1	28°14′47″	91°47′44″	4387	86.0	7.5	41.5	0.4	2.9	383.4	362.7	1.4	263.1	47.6	19.8	239.5	1.3	0.7
	QZM_2	28°14′47″	91°47′44″	4388	82.5	7.5	43.8	1.4	0.0	401.7	348.2	15.7	406.4	46.4	60.4	230.9	1.5	0.3
	QZM_3	28°14′47″	91°47′44″	4388	63.0	6.8	45.3	12.6	1.6	312.3	338.8	15.4	274.7	46.5	22.5	315.9	1.3	0.6
	QZM_4	28°14′55″	91°48′13″	4450	62.0	6.5	30.3	1.4	77.6	277.7	291.2	0.0	291.2	47.9	38.3	316.1	1.3	0.1
	QZM_5	28°14′55″	91°48′14″	4450	56.0	6.8	50.6	1.3	170.0	273.7	284.0	11.7	284.0	60.1	48.3	536.5	1.5	0.3
	QZM_6	28°14′55″	91°48′14″	4450	54.0	7.0	27.0	3.5	65.8	282.1	297.2	0.0	297.2	63.5	47.7	431.8	1.4	0.3
	QZM_7	28°14′55″	91°48′14″	4450	52.0	6.8	28.0	8.4	1.4	285.9	292.4	12.1	292.4	65.9	38.1	143.0	1.1	0.2
	QZM_9	28°14′53″	91°48′31″	4505	67.0	7.0	36.7	1.3	0.1	212.5	461.2	0.0	187.9	21.2	33.7	264.6	1.2	0.9
	QZM_10	28°14′53″	91°48′31″	4505	65.0	7.0	33.6	2.0	0.3	128.6	252.7	0.0	128.3	17.5	18.4	138.3	0.7	0.6
	QZM_11	28°14′53″	91°48′31″	4505	64.0	6.8	20.6	0.0	0.0	214.2	417.9	0.0	202.1	24.5	39.6	328.0	1.2	0.6
	QZM_12	28°14′53″	91°48′31″	4505	62.0	6.8	48.8	1.2	0.0	208.8	381.5	0.0	210.3	30.5	30.9	252.9	1.1	0.5
	QZM_13	28°14′55″	91°48′12″	4438	65.7	6.7	958.6	1.0	0.0	208.4	350.2	0.0	162.0	19.8	26.6	188.9	1.0	0.3
	QZM_14	28°15′50″	91°48′35″	4502	67.5	6.5	111.6	0.9	0.8	412.8	354.7	14.5	346.7	47.6	27.4	231.7	1.4	2.4
	QZM_15	28°14′47″	91°47′44″	4388	78.0	7.0	26.6	0.9	0.2	188.7	467.8	0.0	199.3	25.6	34.9	316.8	1.2	0.2
	QZM_16	28°14′47″	91°47′44″	4388	82.0	6.8	15.6	12.5	0.0	105.9	254.4	0.0	112.6	23.8	39.8	259.1	0.8	0.4
	QZM_17	28°14′50″	91°48′35″	4502	64.6	6.5	3.5	0.7	1.9	209.0	345.4	1.8	170.3	32.7	21.6	264.2	1.0	2.6
	QZM_18	28°14′54″	91°48′32″	4499	62.9	7.0	7.8	1.8	2.2	208.4	383.6	1.0	181.8	35.5	21.6	246.7	1.1	1.2
	QZM_19	28°14′54″	91°48′17″	4448	56.9	7.0	8.1	3.8	2.4	173.5	429.6	1.0	195.2	33.6	26.1	236.3	1.1	0.1
	QZM_20	28°14′56″	91°48′14″	4450	76.9	7.0	3.9	9.9	2.5	245.1	439.4	1.1	237.8	36.4	21.7	295.6	1.3	0.8
	QZM_21	28°14′55	91°48′13″	4450	63.0	7.0	3.6	1.1	2.9	255.7	438.5	1.1	228.4	39.5	20.8	219.1	1.2	0.0
	QZM_22	28°14′55″	91°48′12″	4438	60.3	7.0	2.7	2.2	2.8	278.4	435.8	1.2	239.3	38.4	22.0	285.8	1.3	0.1
	QZM_23	28°14′47″	91°47′44″	4387	66.0	6.5	7.0	0.8	2.9	383.4	362.7	1.4	263.1	47.6	19.8	239.5	1.3	0.4
	QZM_24	28°14′47″	91°47′44″	4388	74.9	7.5	8.8	10.2	2.8	385.2	362.2	1.4	285.9	50.0	20.1	290.8	1.4	0.3
DGJ zone	DGJ_1	29°36′50″	85°44′54″	5067	82.0	8.0	90.5	0.9	20.1	156.8	83.5	15.9	658.7	55.9	11.3	51.3	1.0	0.1
	DGJ_2	29°36′54″	85°45′03″	5070	72.9	7.5	293.1	0.2	10.8	156.4	113.3	10.9	504.5	31.0	6.0	37.2	0.9	0.0
	DGJ_3	29°36′55″	85°45′03″	5070	60.0	7.4	51.8	0.2	9.5	147.8	82.0	10.4	572.9	44.7	4.9	26.6	0.9	0.0
	DGJ_4	29°36′55″	85°45′03″	5070	45.5	7.5	60.7	0.6	81.9	155.9	76.5	9.9	772.3	74.1	5.1	49.7	1.1	0.0
	DGJ_5	29°36′55″	85°45′03″	5070	32.2	7.4	30.9	0.5	133.5	167.9	47.9	9.6	1055.8	126.1	8.9	91.9	1.5	0.0
	DGJ_6	29°36′14″	85°44′50″	5076	78.5	8.0	20.7	0.1	23.1	162.5	109.4	10.5	492.4	30.6	3.2	12.6	0.8	0.0
	DGJ_8	29°36′14″	85°44′49″	5079	80.2	9.5	28.0	0.1	22.2	159.8	84.2	10.1	518.4	31.0	3.4	13.3	0.8	0.0
	DGJ_9	29°36′14″	85°44′49″	5079	70.0	9.5	24.2	0.0	23.2	162.1	82.9	10.2	512.7	34.4	3.3	14.2	0.8	0.0
	DGJ_10	29°36′14″	85°44′49″	5079	60.4	9.0	34.6	0.7	20.7	164.0	88.0	10.9	799.9	67.8	3.4	23.5	1.2	0.0
	DGJ_11	29°36′14″	85°44′49″	5079	45.0	8.8	9.7	0.2	46.0	161.2	84.2	10.1	583.0	56.1	3.4	15.5	0.9	0.0
	DGJ_12	29°36′17″	85°44′49″	5082	81.5	8.0	27.2	0.3	20.3	168.2	89.8	10.1	493.9	28.3	3.5	17.2	0.8	0.0
	DGJ_13	29°36′17″	85°44′49″	5082	66.0	7.8	14.6	0.3	18.1	166.0	84.8	10.2	505.7	30.7	3.1	55.7	0.9	0.0
	DGJ_14	29°36′17″	85°44′49″	5082	50.0	8.0	7.8	1.2	59.7	156.2	8.4	0.0	659.3	70.7	3.6	20.4	0.9	0.2
	DGJ_15	29°36′17″	85°44′49″	5082	42.5	8.0	13.2	2.3	30.2	141.7	72.4	0.0	510.8	65.3	3.8	14.6	0.8	0.0
	DGJ_16	29°36′17″	85°44′49″	5082	35.0	7.8	9.7	2.3	69.5	160.1	62.4	9.5	817.7	89.9	3.5	21.9	1.2	0.0
	DGJ_17	29°36′11″	85°44′52″	5084	36.7	3.0	0.0	0.7	0.4	1.7	104.7	0.0	6.3	3.7	0.9	9.6	0.1	2.0

Sampling Zone	Sample ID	Latitude	Longitude	Elevation (meters about sea level)	Temp. (ºC)	pH	DOC (mg/L)	TOC (%)	F (mg/L)	Cl (mg/L)	SO4 (mg/L)	Br (mg/L)	Na (mg/L)	K (mg/L)	Mg (mg/L)	Ca (mg/L)	Salinity (g/L)	Fe2 (mg/L)
	DGJ_18	29°36′10″	85°44′51″	5075	69.1	4.5	2.3	0.3	0.2	1.3	144.9	0.0	8.4	2.4	1.3	20.2	0.2	0.1
	DGJ_19	29°36′14″	85°44′50″	5076	78.9	8.3	1.5	0.0	26.3	162.3	90.1	0.9	482.4	56.9	0.7	33.9	0.8	0.2
	DGJ_20	29°36′14″	85°44′49″	5079	74.1	7.0	3.4	0.0	24.7	152.0	87.0	0.8	432.3	53.7	0.9	26.0	0.8	0.1
	DGJ_21	29°36′14″	85°44′49″	5073	82.1	7.0	3.4	0.0	24.1	148.7	88.0	0.9	451.2	53.0	0.8	23.5	0.8	0.2
	DGJ_22	29°36′17″	85°44′49″	5082	75.5	6.9	3.3	0.3	24.8	155.3	84.8	0.9	436.5	51.2	0.3	7.2	0.7	0.0
	DGJ_23	29°36′23″	85°44′53″	5072	41.5	6.9	5.5	0.1	26.2	147.9	76.2	0.8	411.0	43.9	0.6	21.1	0.7	0.1
	DGJ_24	29°36′23″	85°44′47″	5079	81.9	7.4	3.6	1.1	24.9	153.9	84.3	0.9	442.8	54.2	1.3	34.0	0.8	0.2
	DGJ_25	29°36′19″	85°44′47″	5079	71.4	7.8	3.6	0.1	25.1	155.3	85.2	0.9	461.1	54.7	0.2	6.0	0.8	0.0
	DGJ_26	29°36′19″	85°44′47″	5079	66.7	7.4	4.2	0.1	25.5	157.9	87.0	1.0	473.1	56.0	0.2	4.7	0.8	0.0
	DGJ_27	29°36′19″	85°44′47″	5079	62.3	7.3	3.5	1.6	25.5	158.5	87.8	0.9	430.5	51.8	0.3	7.6	0.7	0.1
	DGJ_28	29°36′23″	85°44′50″	5079	79.9	7.0	3.2	1.1	25.8	159.4	86.6	0.9	445.5	52.1	0.4	15.2	0.8	0.1
	DGJ_29	29°36′29″	85°44′52″	5077	77.8	7.0	3.2	0.0	21.7	144.8	77.6	0.8	407.7	46.0	0.2	6.1	0.7	0.0
	DGJ_30	29°36′6″	85°45′2″	5061	80.2	6.0	4.3	0.9	18.5	117.9	65.8	1.1	309.3	35.9	0.3	8.6	0.5	0.1
QC zone	QC_1	30°39′59″	91°35′28″	4497	74.2	7.1	4.0	0.9	3.6	439.2	17.4	0.9	331.5	92.9	9.4	173.9	1.1	0.4
	QC_2	30°39′59″	91°35′28″	4497	67.0	7.5	5.2	8.1	3.6	428.8	17.1	0.9	305.1	88.0	8.5	68.0	0.9	0.1
	QC_3	30°39′59″	91°35′29″	4497	61.4	6.9	4.6	0.1	3.5	444.2	17.2	0.9	333.0	94.7	9.5	157.8	1.1	0.4
	QC_4	30°40′1″	91°35′28″	4500	69.5	6.8	4.0	8.8	3.6	423.1	16.9	0.8	359.7	81.0	9.4	96.2	1.0	0.1
	QC_5	30°38′54″	91°35′44″	4495	40.8	7.0	4.5	4.8	3.1	357.2	15.7	0.8	266.2	82.3	10.6	148.0	0.9	0.3
SM zone	SM_1	29°36′6″	86°24′17″	4263	85.9	7.2	7.8	0.7	6.9	172.9	859.8	1.0	227.6	45.5	21.1	308.6	1.6	0.2
	SM_2	29°12′56″	86°24′17″	4263	79.5	7.2	2.2	0.1	5.4	132.0	1216.6	0.9	239.5	47.3	17.2	311.2	2.0	0.7
	SM_3	29°12′56″	86°24′17″	4263	73.0	3.4	0.5	2.8	10.2	146.9	1365.0	0.8	198.0	47.4	19.3	449.0	2.2	31.6
	SM_4	29°12′58″	86°24′14″	4262	84.9	8.5	5.7	4.8	19.0	751.8	29.2	2.3	945.5	161.8	1.4	15.8	1.9	0.2
GD zone	GD_1	28°31′13″	91°52′56″	4516	70.9	7.0	3.6	0.2	9.8	558.1	168.6	1.8	0.1	79.6	0.6	16.9	0.8	0.0
	GD_2	28°31′13″	91°52′56″	4516	61.8	7.0	4.2	0.2	9.7	648.0	164.3	2.0	0.0	91.1	4.4	54.7	1.0	0.1
	GD_3	28°31′13″	91°52′56″	4516	53.9	7.5	3.7	0.5	9.8	662.1	168.1	2.0	0.0	90.3	4.4	52.6	1.0	0.1
	GD_4	28°31′13″	91°52′56″	4516	45.7	7.5	3.7	1.2	9.9	670.7	171.7	2.1	0.0	97.1	4.7	53.5	1.0	0.1
	GD_5	28°31′11″	91°52′59″	4495	78.4	8.0	4.2	1.7	10.0	677.3	173.7	2.1	0.0	85.9	4.1	45.4	1.0	0.1

Table S2. Affiliation of the identified phyla from the obtained 16S rRNA gene sequence reads of the total, abundant, intermediate and rare biospheres (highlighted with grey background means phyla common in the abundant, intermediate and rare biosphere)

Total biosphere Phyla	Relative abundance (%)	Phyla in abundant biosphere	Relative abundance (%)	Phyla in intermediate biosphere	Relative Abundance (%)	Phyla in rare biosphere	Relative abundance (%)
Chloroflexi	17.00	*Chloroflexi*	17.81	*Thermotogae*	5.60	*Dictyoglomi*	4.33
Proteobacteria	12.94	*Proteobacteria*	13.58	*Armatimonadetes*	4.49	*Hydrogenedentes*	4.20
Deinococcus-Thermus	10.23	*Deinococcus-Thermus*	10.73	*Thermodesulfobacerteria*	4.41	*Atribacteria*	4.05
Bacteroidetes	8.59	*Aquificae*	8.88	*Bacteroidetes*	4.41	*Hadesarchaea*	4.02
Aquificae	8.40	*Bacteroidetes*	8.62	*Ignavibacteriae*	4.12	*Aminicenantes*	3.73
Firmicutes	4.67	*Firmicutes*	4.78	*Acetothermia*	3.81	*Microgenomates*	3.55
Cyanobacteria	4.56	*Cyanobacteria*	4.66	*Chlorobi*	3.64	*Calescamantes*	3.51
Acetothermia	4.11	*Acetothermia*	4.13	*Planctomycetes*	3.56	*Omnitrophica*	2.57
Thermodesulfobacteria	2.93	*Thermodesulfobacteria*	2.86	*Verrucomicrobia*	3.50	*Altiarchaeales*	2.45
Thermotogae	2.30	*Thermotogae*	2.12	*Acidobacteria*	3.32	*Chlamydiae*	2.43
Crenarchaeota	1.73	*Crenarchaeota*	1.65	*Nitrospirae*	3.23	*Crenarchaeota*	2.11
Chlorobi	1.48	*Chlorobi*	1.37	*Crenarchaeota*	3.16	*Fervidibacteria*	2.10
Bathyarchaeota	1.37	*Bathyarchaeota*	1.34	*Parcubacteria*	3.13	*Woesearchaeota*(DHVE	1.99
Parcubacteria	1.36	*Parcubacteria*	1.27	*Aquificae*	3.10	*Bathyarchaeota*	1.70
Nitrospirae	1.27	*Nitrospirae*	1.67	*Fervidibacteria*	3.06	*Lentisphaerae*	1.68
Ignavibacteriae	1.06	Others	15.23	*Cyanobacteria*	2.72	*Actinobacteria*	1.66
Fervidibacteria	1.02			*Spirochaetae*	2.65	*Tenericutes*	1.65
Others	14.93			*Firmicutes*	2.48	*Caldiserica*	1.58
				Thaumarchaeota	2.46	*Verrucomicrobia*	1.55
				Euryarchaeota	2.18	*Korarchaeota*	1.45
				Bathyarchaeota	2.01	*Planctomycetes*	1.26

Total biosphere Phyla	Relative abundance (%)	Phyla in abundant biosphere	Relative abundance (%)	Phyla in intermediate biosphere	Relative Abundance(%)	Phyla in rare biosphere	Relative abundance (%)
				Aigarchaeota	1.80	*Fibrobacteres*	1.20
				Caldiserica	1.55	*Chlorobi*	1.07
				Chloroflexi	1.55	*Thaumarchaeota*	1.04
				Actinobacteria	1.52	*Acidobacteria*	1.01
				Others	22.54	Others	42.13

Table S3 Affiliation of the identified Class from the obtained 16S rRNA gene sequence reads of the total, abundant, intermediate and rare biospheres

Total biosphere Class	Relative abundance (%)	Abundant biosphere Class	Relative abundance (%)	Intermediate biosphere Class	Relative abundance (%)	Rare biosphere Class	Relative abundance (%)
Deinococci	10.23	*Deinococci*	13.46	*Thermotogae*	4.74	*Acidimicrobiia*	2.28
Chloroflexia	9.78	*Chloroflexia*	12.81	*Thermodesulfobacteria*	3.74	*Opitutae*	2.20
Aquificae	8.59	*Aquificae*	11.13	*Gammaproteobacteria*	3.50	*Bacteroidetes Incertae Sedis*	2.15
Betaproteobacteria	5.40	*Betaproteobacteria*	6.92	*Ignavibacteria*	3.49	*Coriobacteriia*	2.11
Sphingobacteriia	4.77	*Sphingobacteriia*	6.06	*Solibacteres*	3.49	*Dictyoglomia*	2.06
Cyanobacteria	4.56	*Cyanobacteria*	5.85	*Alphaproteobacteria*	3.22	*Atribacteria Incertae Sedis*	1.92
Gammaproteobacteria	4.39	*Gammaproteobacteria*	5.53	*Chlorobia*	3.09	*Fimbriimonadia*	1.84
Bacilli	3.01	*Bacilli*	3.88	*Sphingobacteriia*	3.02	Chthonomonadetes	1.75
Anaerolineae	2.94	*Anaerolineae*	3.72	*Planctomycetacia*	2.95	*Thermomicrobia*	1.75
Thermodesulfobacteria	2.93	*Thermodesulfobacteria*	3.58	*Nitrospira*	2.73	*Candidatus Roizmanbacteria*	1.73
Thermotogae	2.30	*Thermotogae*	2.66	*Betaproteobacteria*	2.68	*Caldilineae*	1.73
Thermoprotei	1.73	*Thermoprotei*	2.07	*Thermoprotei*	2.67	*Verrucomicrobiae*	1.70
Clostridia	1.61	*Clostridia*	1.98	*Aquificae*	2.63	*Thermoleophilia*	1.64
Chlorobia	1.48	*Chlorobia*	1.72	*Deltaproteobacteria*	2.27	*Methanobacteria*	1.63
Alphaproteobacteria	1.43	*Alphaproteobacteria*	1.63	*Spirochaetes*	2.24	*Candidatus Azambacteria*	1.50
Deltaproteobacteria	1.31	*Deltaproteobacteria*	1.56	*Cyanobacteria*	2.23	*Acidobacteria*	1.24
Nitrospira	1.27	*Nitrospira*	1.46	*Anaerolineae*	2.08	*Spartobacteria*	1.21
Ignavibacteria	1.06	*Ignavibacteria*	1.13	*Cytophagia*	1.93	*Chlamydiae*	1.15
Others	30.85	Others	12.68	*Clostridia*	1.93	*Candidatus Woesebacteria*	1.06
				Phycisphaerae	1.76	*Thaumarchaeota Incertae Sedis*	1.05
				Others	43.62	Others	66.30

Table S4. Affiliation of the identified phyla from the obtained 16S rRNA gene sequence reads of the total biosphere in the moderate- and high-temperature hot springs

Phyla identified in the moderate-T hot springs	Relative abundance (%)	Phyla identified in the high-T hot springs	Relative abundance (%)
Chloroflexi	26.96	*Aquificae*	20.74
Bacteroidetes	18.03	*Proteobacteria*	16.56
Proteobacteria	13.76	*Bacteroidetes*	14.98
Aquificae	8.63	*Deinococcus-Thermus*	14.32
Cyanobacteria	8.21	*Firmicutes*	13.69
Bathyarchaeota	8.19	*Chloroflexi*	12.36
Deinococcus-Thermus	8.16	*Thermotogae*	11.84
Acetothermia	7.52	*Thermodesulfobacteria*	9.47
Fervidibacteria	6.76	*Acetothermia*	9.47
Euryarchaeota	6.58	*Dictyoglomi*	9.44
Thermodesulfobacteria	4.70	*Bathyarchaeota*	8.01
Woesearchaeota (DHVEG-6)	4.57	*Cyanobacteria*	7.18
Altiarchaeales	4.35	*Parcubacteria*	6.86
Chlorobi	4.14	*Crenarchaeota*	5.83
Armatimonadetes	3.75	*Chlorobi*	5.22
Nitrospirae	3.59	*Fervidibacteria*	5.16
Crenarchaeota	3.51	*Spirochaetae*	4.71
Deferribacteres	3.49	*Ignavibacteriae*	4.03
Thaumarchaeota	3.34	*Woesearchaeota* (DHVEG-6)	3.75
Firmicutes	3.26	*Atribacteria*	3.23
Spirochaetae	2.98	*Planctomycetes*	3.04
Verrucomicrobia	2.88	*Euryarchaeota*	2.98
Acidobacteria	2.83	*Thaumarchaeota*	2.94
Planctomycetes	2.79	*Verrucomicrobia*	2.81
Thermotogae	2.76	*Acidobacteria*	2.50
Microgenomates	2.41	*Actinobacteria*	1.78
Ignavibacteriae	2.40	*Aigarchaeota*	1.71
Parcubacteria	2.17	*Nitrospirae*	1.55
Caldiserica	1.42	*Calescamantes*	1.40
Aigarchaeota	1.23	Others	8.54
Gracilibacteria	1.22		
Aminicenantes	1.05		
Others	14.37		

Table S5. Affiliation of the identified phyla from the obtained 16S rRNA gene sequence reads of the abundant biosphere in the moderate and high-temperature hot springs

Phyla identified in the moderate-T hot springs	Relative abundance (%)	Phyla identified in the high-T hot springs	Relative abundance (%)
Chloroflexi	16.54	*Aquificae*	5.74
Proteobacteria	8.14	*Proteobacteria*	5.51
Deinococcus-Thermus	6.03	*Deinococcus-Thermus*	4.73
Bacteroidetes	5.66	*Firmicutes*	3.68
Cyanobacteria	4.27	*Bacteroidetes*	3.29
Aquificae	3.14	*Chloroflexi*	1.92
Acetothermia	2.58	*Thermotogae*	1.70
Thermodesulfobacteria	1.43	*Acetothermia*	1.54
Bathyarchaeota	1.21	*Thermodesulfobacteria*	1.42
Nitrospirae	1.14	*Crenarchaeota*	1.11
Firmicutes	1.10	*Fervidibacteria*	0.85
Chlorobi	1.08	*Cyanobacteria*	0.57
Acidobacteria	0.79	*Euryarchaeota*	0.55
Thaumarchaeota	0.78	*Parcubacteria*	0.50
Parcubacteria	0.78	Others	1.99
Woesearchaeota (DHVEG-6)	0.71		
Ignavibacteriae	0.61		
Armatimonadetes	0.55		
Crenarchaeota	0.54		
Planctomycetes	0.47		
Thermotogae	0.45		
Others	1.92		

Table S6. Affiliation of the identified phyla from the obtained 16S rRNA gene sequence reads of the intermediate biosphere in the moderate and high-temperature hot springs

Phyla identified in the moderate-T hot springs	Relative abundance (%)	Phyla identified in the high-T hot springs	Relative abundance (%)
Thermotogae	6.14	*Thermotogae*	4.56
Armatimonadetes	5.01	*Thermodesulfobacteria*	4.12
Ignavibacteriae	4.99	*Chloroflexi*	3.73
Bacteroidetes	4.81	*Acetothermia*	3.65
Verrucomicrobia	4.72	*Bacteroidetes*	3.63
Planctomycetes	4.65	*Armatimonadetes*	3.50
Chlorobi	4.61	*Thaumarchaeota*	3.39
Thermodesulfobacteria	4.57	*Fervidibacteria*	3.34
Acidobacteria	4.12	*Crenarchaeota*	3.19
Aquificae	3.90	*Cyanobacteria*	2.98
Acetothermia	3.89	*Parcubacteria*	2.83
Spirochaetae	3.55	*Nitrospirae*	2.82
Nitrospirae	3.44	*Bathyarchaeota*	2.79
Parcubacteria	3.28	*Ignavibacteriae*	2.49
Crenarchaeota	3.14	*Dictyoglomi*	2.16
Firmicutes	2.98	*Acidobacteria*	1.83
Fervidibacteria	2.90	*Chlorobi*	1.83
Euryarchaeota	2.87	*Atribacteria*	1.61
Cyanobacteria	2.57	*Aquificae*	1.61
Aigarchaeota	2.09	*Firmicutes*	1.54
Caldiserica	1.98	*Planctomycetes*	1.52
Thaumarchaeota	1.96	*Actinobacteria*	1.43
Woesearchaeota (DHVEG-6)	1.78	*Proteobacteria*	1.36
Aminicenantes	1.71	*Aigarchaeota*	1.27
Omnitrophica	1.69	*Verrucomicrobia*	1.23
Microgenomates	1.65	Others	13.49
Bathyarchaeota	1.59		
Actinobacteria	1.57		
Altiarchaeales	1.24		
Deferribacteres	1.14		
Others	17.72		

Table S7. Affiliation of the identified phyla from the obtained 16S rRNA gene sequence reads of the rare biosphere in the moderate- and high-temperature hot springs

Phyla identified in the moderate-T hot springs	Relative abundance (%)	Phyla identified in the high-T hot springs	Relative abundance (%)
Atribacteria	3.24	*Aminicenantes*	1.60
Hadesarchaea	3.11	*Hydrogenedentes*	1.37
Dictyoglomi	3.00	*Dictyoglomi*	1.33
Calescamantes	2.96	*Microgenomates*	1.31
Hydrogenedentes	2.83	*Caldiserica*	1.28
Microgenomates	2.24	*Bathyarchaeota*	1.12
Aminicenantes	2.13	*Verrucomicrobia*	0.93
Chlamydiae	1.99	*Hadesarchaea*	0.91
Crenarchaeota	1.94	*Planctomycetes*	0.90
Fervidibacteria	1.75	*Actinobacteria*	0.90
Omnitrophica	1.74	*Omnitrophica*	0.83
Altiarchaeales	1.69	*Tenericutes*	0.83
Lentisphaerae	1.29	*Atribacteria*	0.81
Woesearchaeota (DHVEG-6)	1.20	*Woesearchaeota (DHVEG-6)*	0.78
Lokiarchaeota	0.97	*Acidobacteria*	0.76
Fibrobacteres	0.95	*Altiarchaeales*	0.76
Korarchaeota	0.83	*Spirochaetae*	0.71
Tenericutes	0.82	*Armatimonadetes*	0.70
Actinobacteria	0.76	*Chlorobi*	0.68
Aenigmarchaeota	0.70	*Korarchaeota*	0.62
Gracilibacteria	0.69	*Ignavibacteriae*	0.60
Parcubacteria	0.63	*Thaumarchaeota*	0.59
Verrucomicrobia	0.62	*Calescamantes*	0.55
Poribacteria	0.62	Others	11.21
Elusimicrobia	0.61		
Aigarchaeota	0.60		
Bathyarchaeota	0.58		
Others	27.42		

Table S8 Identified potential microbial ecological functions and their relative abundance of in the total, abundant, intermediate and rare biosphere of the studied hot springs

Microbial ecological functions	Relative abundance of microbial ecological functions in average (%)			
	Total	Abundant	Intermediate	Rare
Number of identified functions	22	15	21	20
photoautotrophy	6.48	5.43	2.55	3.85
photoheterotrophy	0.02	0.00	0.11	0.14
chemoheterotrophy	7.06	26.27	3.99	13.25
anoxygenic photoautotrophy	0.62	0.20	0.52	0.53
sulfur compounds respiration	5.05	7.86	4.16	3.76
dark sulfur compounds oxidation	6.90	7.36	0.74	0.70
sulfate reduction	3.71	3.04	2.72	1.55
denitrification	2.14	6.97	1.30	2.11
ammonia oxidation	1.27	2.81	0.00	0.00
nitrification	1.27	2.81	1.53	3.13
nitrogen fixation	1.14	4.43	0.98	0.74
dark hydrogen oxidation	0.65	1.37	0.63	0.78
aromatic compound degradation	0.47	0.00	0.30	0.42
fermentation	0.36	1.73	0.78	0.64
knallgas bacteria	0.36	1.21	0.33	0.23
methanogenesis	0.29	0.35	0.10	0.68
xylan degradation	0.21	0.00	0.30	0.22
nitrite respiration	0.17	0.00	0.15	0.00
iron respiration	0.16	0.22	0.17	0.29
methylotrophy	0.02	0.00	0.04	0.44
methanol oxidation	0.02	0.00	0.04	0.23
hydrocarbon degradation	0.02	0.00	0.03	0.15
others	83.41	75.17	78.54	83.41

Table S9 The relative abundance of the identified microbial ecological functions in the studied MT and HT hot springs for the total, abundant, intermediate and rare biospheres

Microbial ecological functions	Average relative abundance (%) of the identified microbial ecological functions							
	**Total**		**Abundant**		**Intermediate**		**Rare**	
	**MT**	**HT**	**MT**	**HT**	**MT**	**HT**	**MT**	**HT**
photoautotrophy	8.85	2.33	6.24	4.02	2.95	1.85	3.29	4.83
photoheterotrophy	0.00	0.00	0.00	0.00	0.02	0.26	0.16	0.11
chemoheterotrophy	4.95	10.75	18.15	40.48	2.38	6.82	11.82	15.76
anoxygenic photoautotrophy	0.86	0.19	0.22	0.15	0.68	0.25	0.35	0.85
sulfur compounds respiration	4.57	5.90	7.03	9.33	4.47	3.60	3.46	4.28
dark sulfur compounds oxidation	6.21	8.11	3.17	14.68	0.54	1.10	0.59	0.89
sulfate reduction	3.46	4.15	2.60	3.81	2.97	2.29	1.50	1.65
denitrification	2.14	2.14	4.53	11.25	1.21	1.46	1.78	2.69
ammonia oxidation	1.87	0.22	2.63	3.13	0.00	0.00	0.00	0.00
nitrification	1.87	0.21	2.63	3.13	2.01	0.70	3.05	3.28
nitrogen fixation	1.20	1.03	4.70	3.95	1.01	0.92	0.60	0.97
dark hydrogen oxidation	0.37	1.13	0.12	3.56	0.49	0.86	0.76	0.82
aromatic compound degradation	0.33	0.71	0.00	0.00	0.21	0.45	0.22	0.77
fermentation	0.17	0.70	1.91	1.41	0.56	1.17	0.47	0.94
knallgas bacteria	0.33	0.41	0.00	3.34	0.25	0.48	0.24	0.23
methanogenesis	0.24	0.38	0.17	0.67	0.15	0.02	0.69	0.67
xylan degradation	0.10	0.41	0.00	0.00	0.08	0.68	0.30	0.07
nitrite respiration	0.11	0.28	0.00	0.00	0.09	0.25	0.00	0.00
iron respiration	0.12	0.24	0.30	0.08	0.15	0.19	0.20	0.45
methylotrophy	0.00	0.05	0.00	0.00	0.03	0.06	0.20	0.86
methanol oxidation	0.00	0.05	0.00	0.00	0.02	0.06	0.10	0.44
hydrocarbon degradation	0.00	0.05	0.00	0.00	0.03	0.03	0.05	0.30

Fig. S1 Ecological characters of the sampled Tibetan hot springs in QZM (A, B, C, D), DGJ (E, F, G, H, I), QC (J), SM (K) and GD (L) zones.

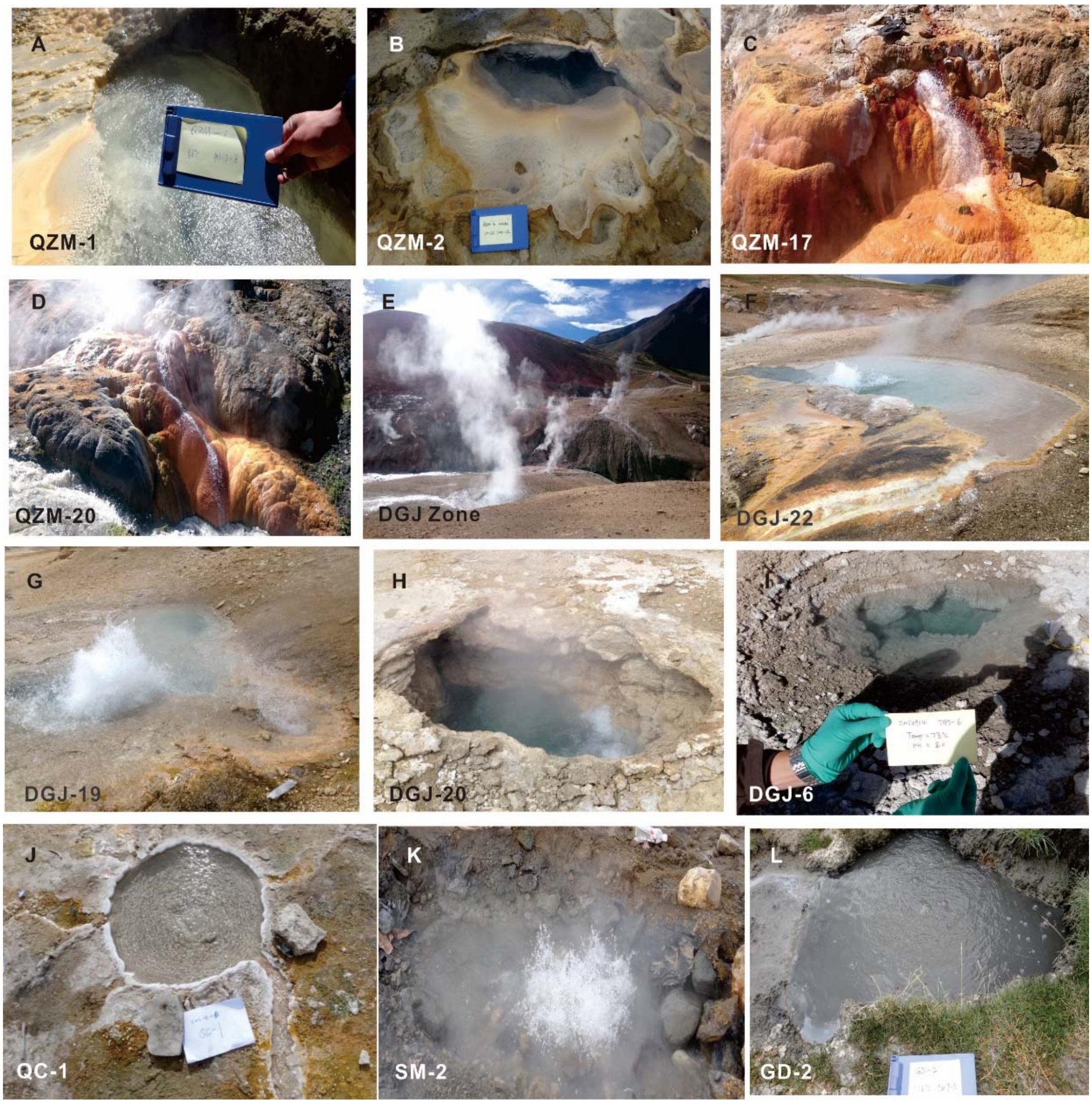

Fig. S2 Geochemistry clustering of the studied hot springs based on Euclidean distance of the measured environmental factors.

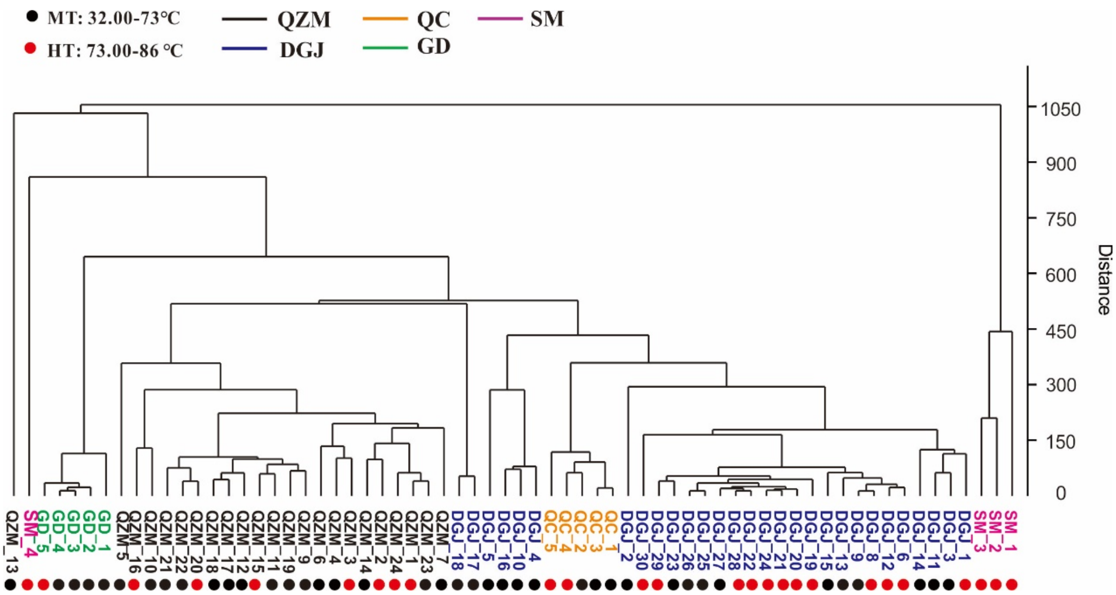

Fig. S3 UPGMA cluster dendrograms based on Bray-Curtis dissimilarities (at the 97% cutoff) of 16S rRNA gene fragments obtained in this study. A, B, C and D panels were for total, abundant (relative abundance > 1%), intermediate (relative abundance 0.01-1%) and rare (relative abundance < 0.01%) OTUs, respectively.

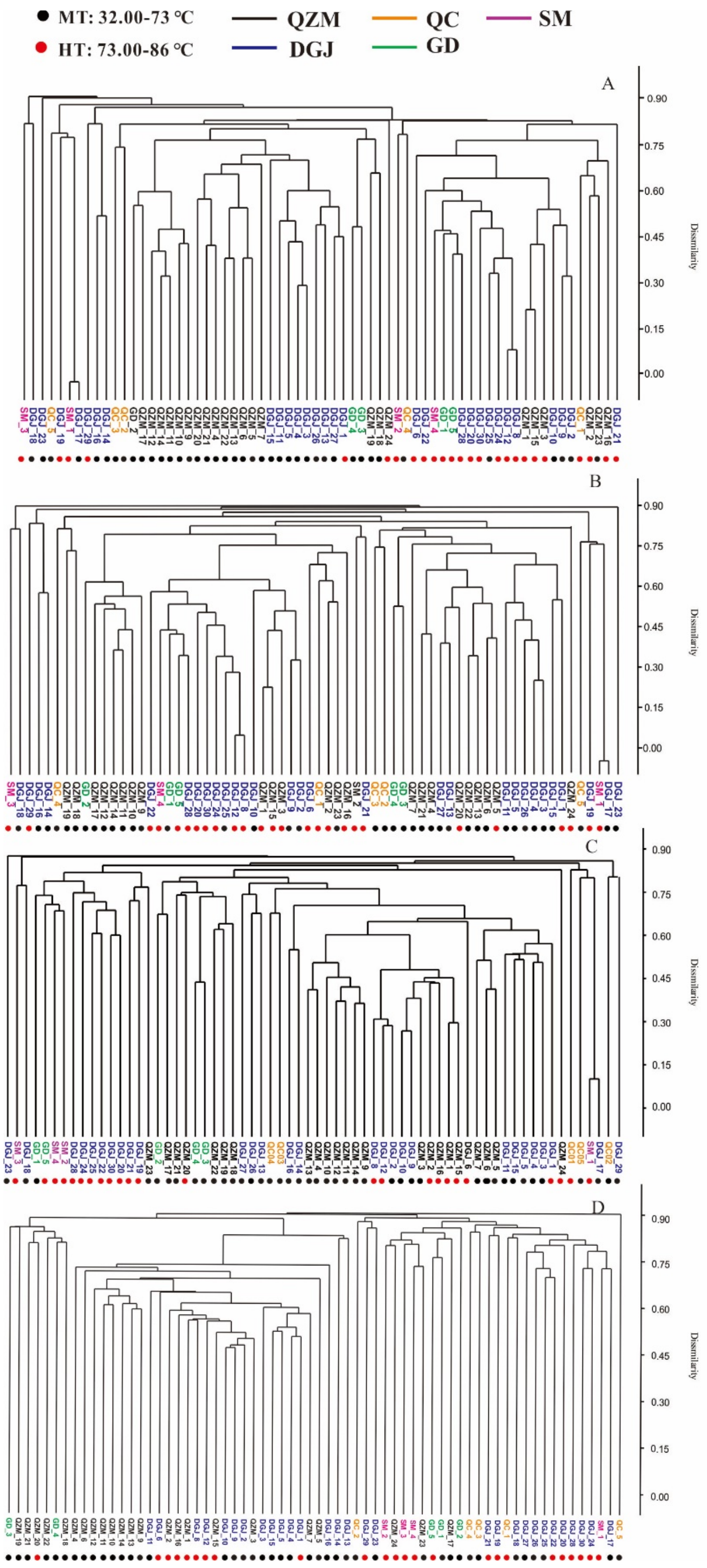

Supplement: Supplementary file 1 [file Data_Sheet_1.pdf]
